# Supplementary material for: Disrupting tumour vasculature and recruitment of aPDL1-loaded platelets control tumour metastasis
Source: Nat Commun. 2021 May 13;12:2773. doi: 10.1038/s41467-021-22674-3 (PMC8119987; doi:10.1038/s41467-021-22674-3)
Supplement: Supplementary file 1 — Supplementary Information [file 41467_2021_22674_MOESM1_ESM.pdf]

Supplementary Information

Disrupting tumour vasculature and recruitment of aPDL1-loaded platelets control tumour metastasis

Hongjun Li[1,2,3,4,5], Zejun Wang[3,4,5], Zhaowei Chen[1,3,6], Tianyuan Ci[3], Guojun Chen[3,4,5], Di Wen[3,4,5], Ruoxin Li[3,5], Jinqiang Wang[1,3,4,5], Huan Meng[5], R Bryan Bell[7], Zhifeng Gu[8], Gianpietro Dotti[9], Zhen Gu[1,2,3,4,5]*

Affiliations:

[1] College of Pharmaceutical Sciences, Zhejiang University, Hangzhou, Zhejiang 310058, China

[2] Zhejiang University Medical Center, Sir Run Run Shaw Hospital, Hangzhou, Zhejiang 310058, China

[3] Department of Bioengineering, University of California, Los Angeles, California 90095, United States

[4] Jonsson Comprehensive Cancer Center, University of California, Los Angeles, California 90095, United States

[5] California NanoSystems Institute, University of California, Los Angeles, California 90095, United States

[6] Institute of Food Safety and Environment Monitoring, College of Chemistry, Fuzhou University, Fuzhou 350108, China

[7] Earle A. Chiles Research Institute in the Robert W. Franz Cancer Center, Providence Cancer Institute, 4805 NE Glisan St. Suite 2N35, Portland, OR 97213, United States

[8] Research Center of Clinical Medicine, Affiliated Hospital of Nantong University, Nantong, 226001, China

[9] Joint Department of Biomedical Engineering, University of North Carolina at Chapel Hill and North Carolina State University, Raleigh, North Carolina 27695, United States

*To whom correspondence should be addressed: Email: guzhen@zju.edu.cn (Z.G.).

This PDF file includes:

Supplementary Figure 1. Preparation route of platelet conjugated with aPDL1.

Supplementary Figure 2. Surface proteins on native platelets and P@aPDL1.

Supplementary Figure 3. aPDL1 release profile from P@PDL1.

Supplementary Figure 4. Biocompatibility of Vidamezan *in vivo*.

Supplementary Figure 5. Metastasis formation *in vivo.*

Supplementary Figure 6. Cell apoptosis in tumour metastases after treatment.

Supplementary Figure 7. The gating strategy for T cell analysis by flow cytometry.

Supplementary Figure 8. Representative flow cytometry analysis of $CD3^+$ T cells.

Supplementary Figure 9. T cell distribution in tumour metastases in the lung parenchyma.

Supplementary Figure 10. Cytotoxic T cells analysis in 4T1 metastatic lesions after treatment.

Supplementary Figure 11 Cytokine detection in metastatic lesions after treatment.

Supplementary Figure 12. Immune response in the non-tumour bearing mice after P@aPDL1 + Vadimezan treatment.

Supplementary Figure 13. The gating strategy to identify T cell memory cells by flow cytometry.

Supplementary Figure 14. $CD3^+$ T cells in the peripheral blood of treated mice.

Supplementary Figure 15. Cytotoxic T cells analysis in B16F10 melanoma metastatic lesions after treatment.

Supplementary Figure 16. Survival of mice bearing metastatic Lewis lung cancer after treatment.

Supplementary Figure 17. PDL1 expression by tumour cells after treatment with Vadimezan.

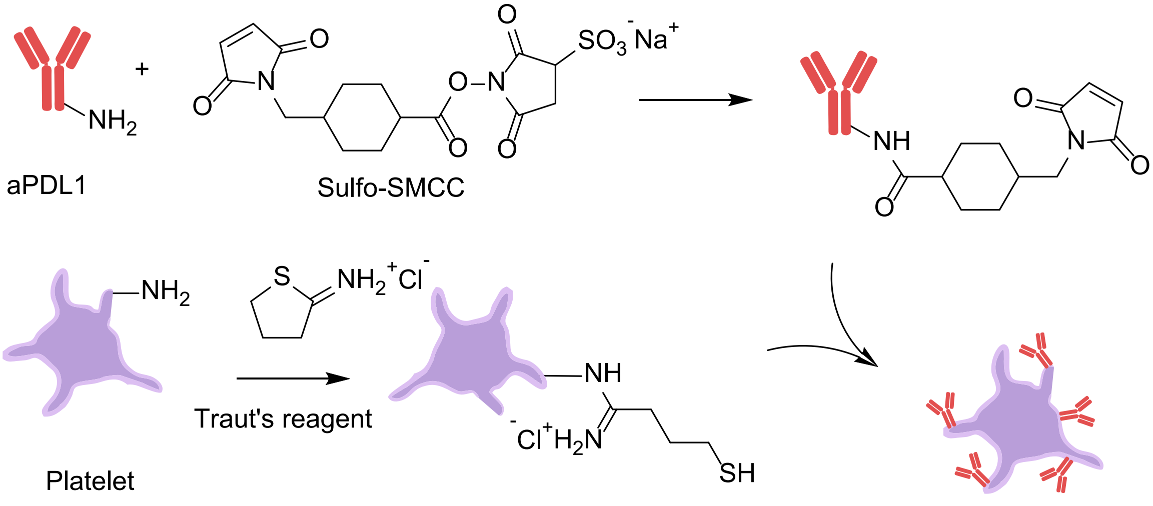

Supplementary Figure 1. Preparation route of platelet conjugated with aPDL1.

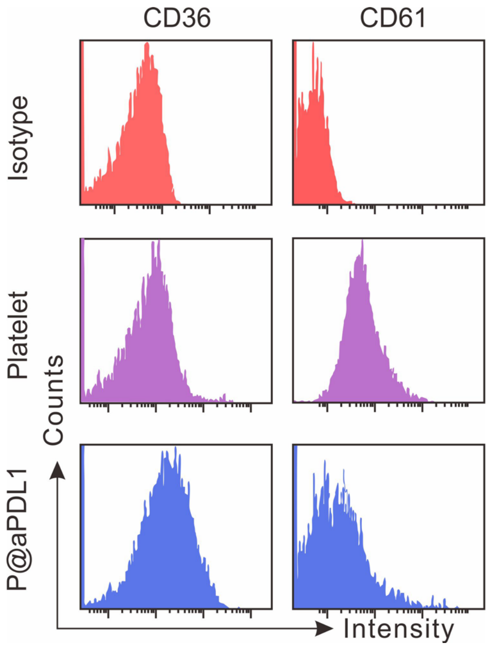

Supplementary Figure 2. Surface proteins on native platelets and P@aPDL1. Flow cytometry analysis of the proteins on native platelets and P@aPDL1. The isotype control antibody was an anti-human CD8 antibody.

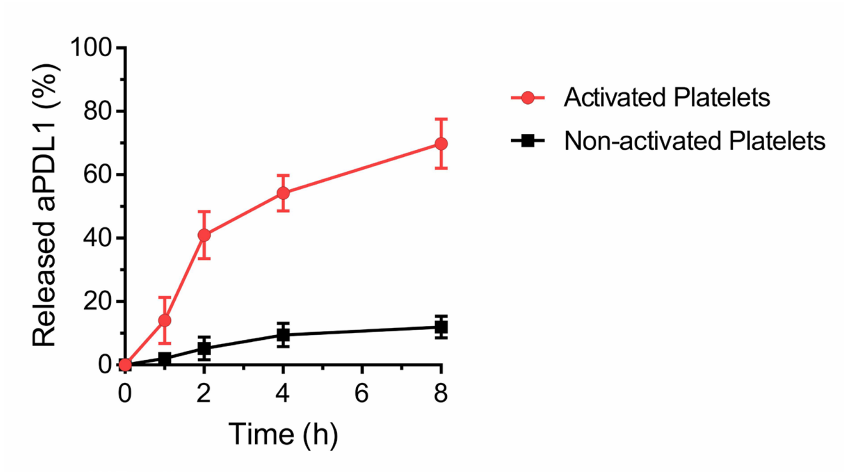

Supplementary Figure 3. aPDL1 release profile from P@PDL1. The release profile of aPDL1 from P@aPDL1 with or without activation by thrombin. Data are presented as mean ± SD, *n* = 3 biologically independent samples.

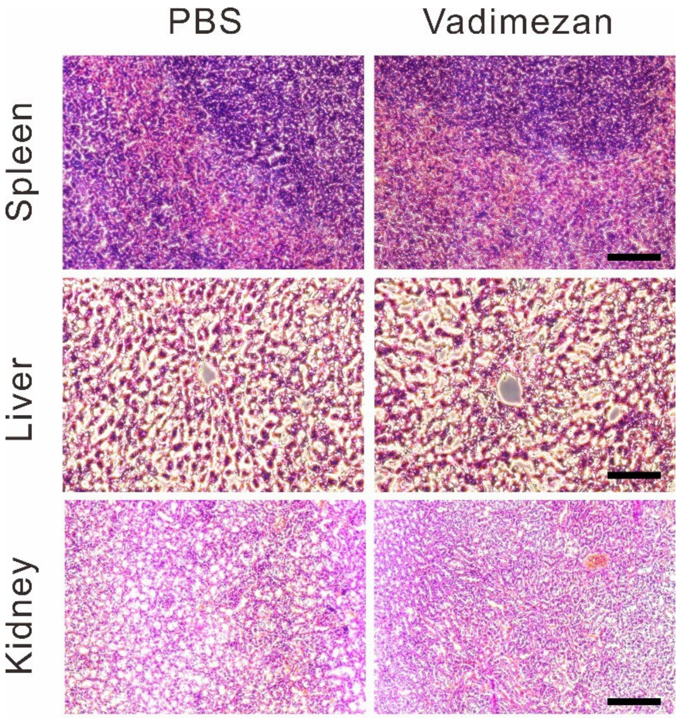

Supplementary Figure 4. Biocompatibility of Vidamezan *in vivo*. The images show the H&E staining of spleen, liver and kidney collected from mice after treatment with PBS or Vadimezan at the dose of 15 mg/kg. Scale bars: 40 μm.

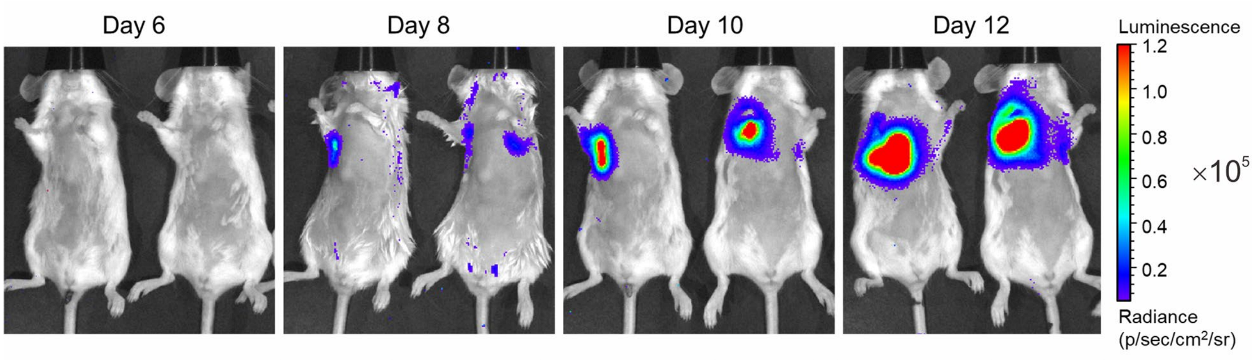

Supplementary Figure 5. Metastasis formation *in vivo*. *In vivo* tumour bioluminescence images of mice at different days after 4T1 tumour cell inoculation intravenously.

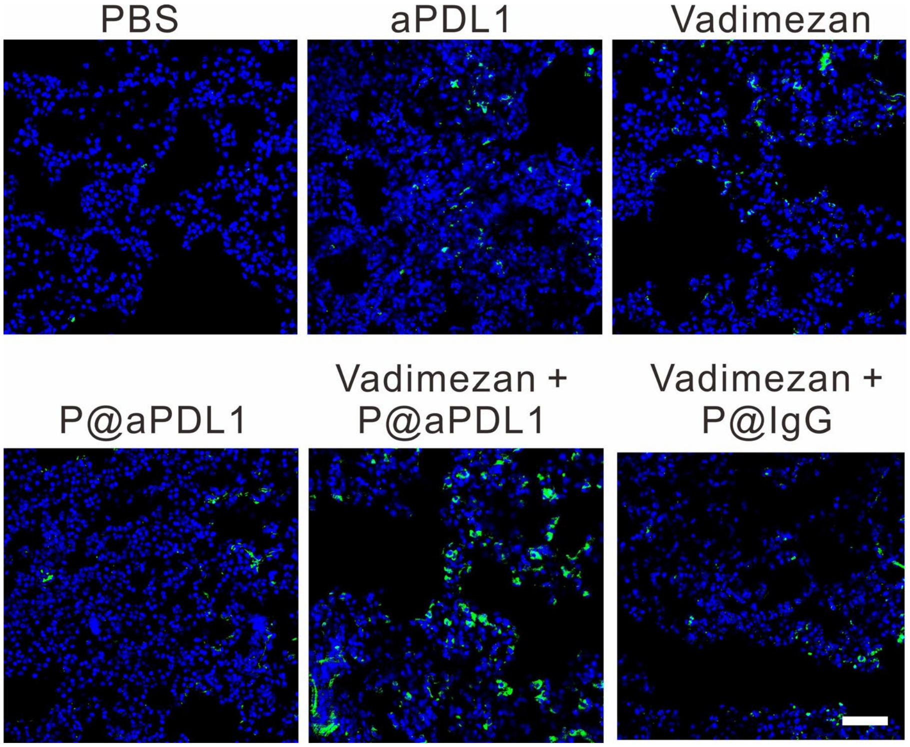

Supplementary Figure 6. Cell apoptosis in tumour metastases after treatment. Confocal images displaying apoptosis of tumour cells in the metastasis in the lung parenchyma after treatment. Apoptotic cells were labeled with Terminal deoxynucleotidyl transferase dUTP nick end labeling (TUNEL) assay. Scale bar: 50 μm.

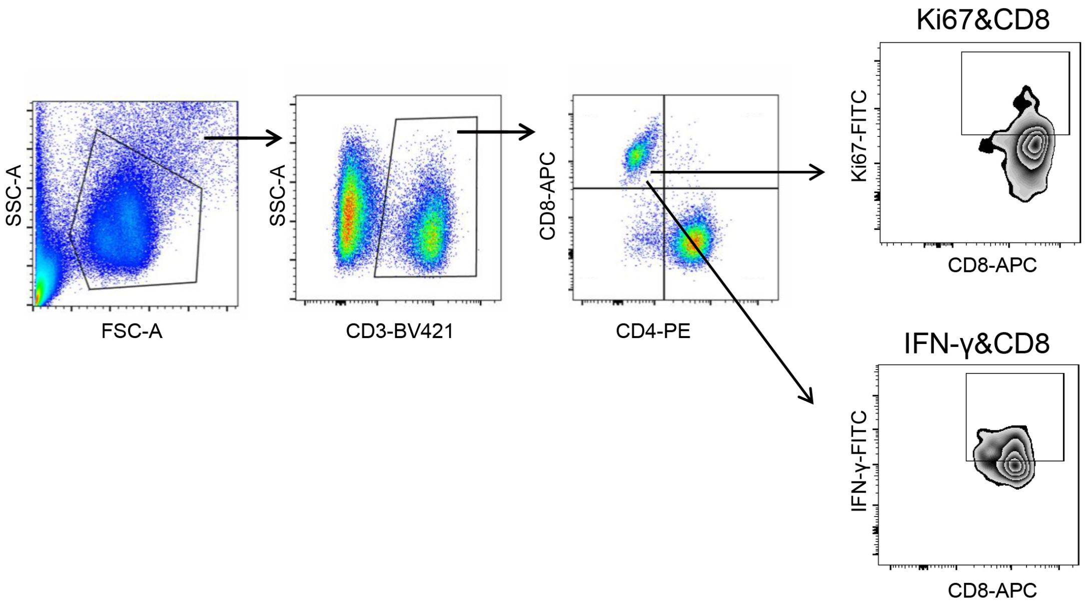

Supplementary Figure 7. Gating strategy for T cell analysis by flow cytometry.

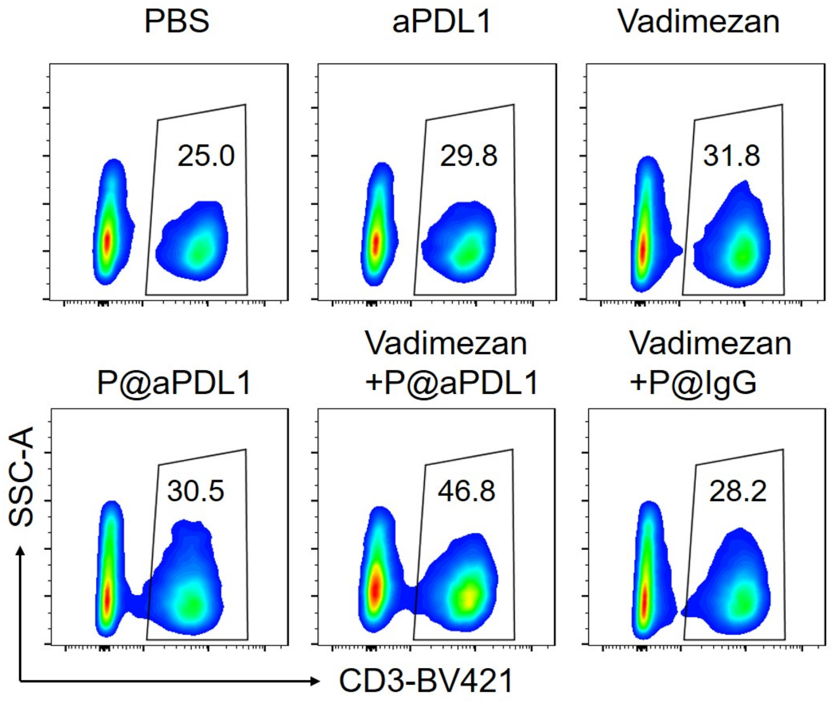

Supplementary Figure 8. Representative flow cytometry analysis of $CD3^+$ T cells.

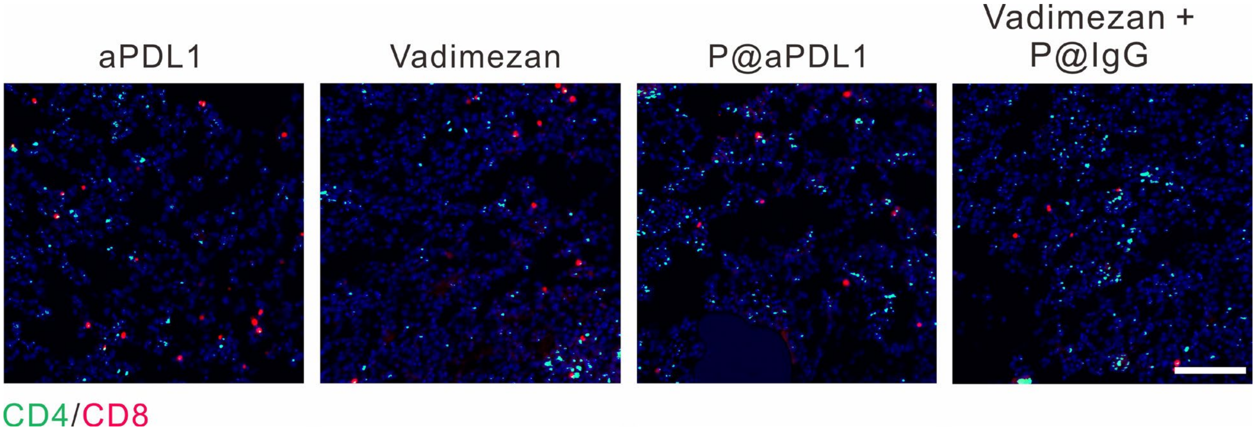

Supplementary Figure 9. T cell distribution in tumour metastases in the lung parenchyma. Confocal images displaying the distribution of CD4/CD8 T cells in the metastatic lesion within the lung. Scale bar: 100 µm.

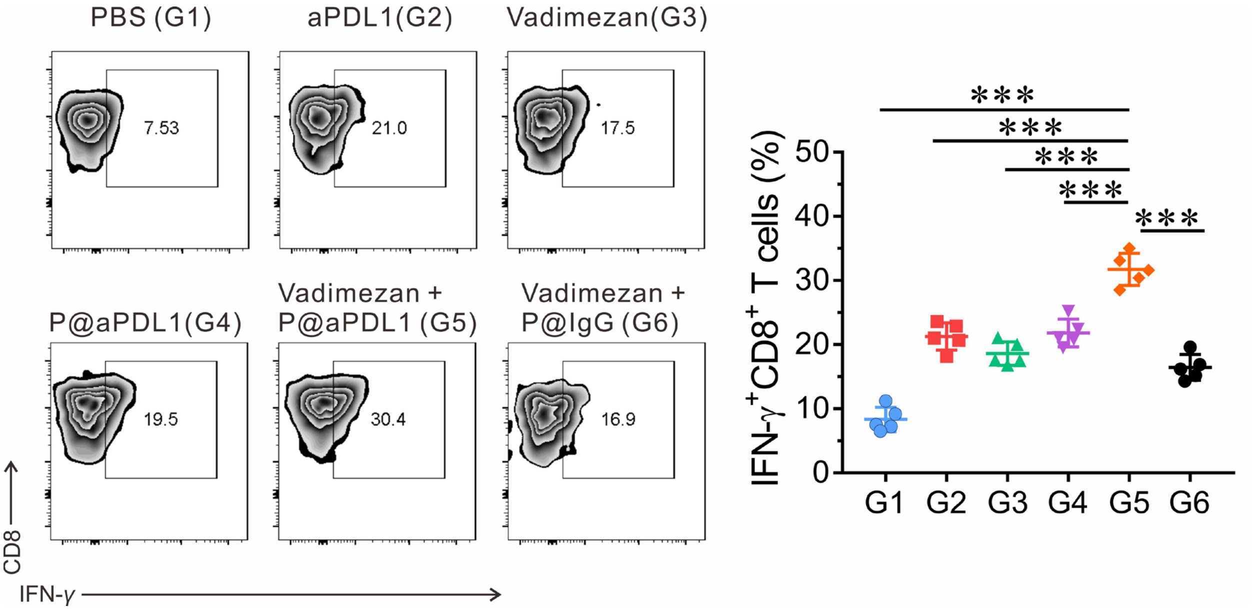

Supplementary Figure 10. Cytotoxic T cells analysis in 4T1 metastatic lesions after treatment. Representative flow cytometry analysis of IFN-γ^{+}CD8^{+} T cells gating on CD3^{+} T cells in the tumor metastases, $n = 5$ biologically independent animals. G1, PBS; G2, aPDL1; G3, Vadimezan; G4, P@aPDL1; G5, P@aPDL1 + Vadimezan; G6, P@IgG + Vadimezan. Data are presented as mean ± SD, statistical significance is calculated *via* one-way ANOVA with a Tukey post-hoc test. P value: *** $P < 0.001$. G1 *vs.* G5: $P < 0.0001$, G2 *vs.* G5: $P < 0.0001$, G3 *vs.* G5: $P < 0.0001$, G4 *vs.* G5: $P < 0.0001$, G6 *vs.* G5: $P < 0.0001$.

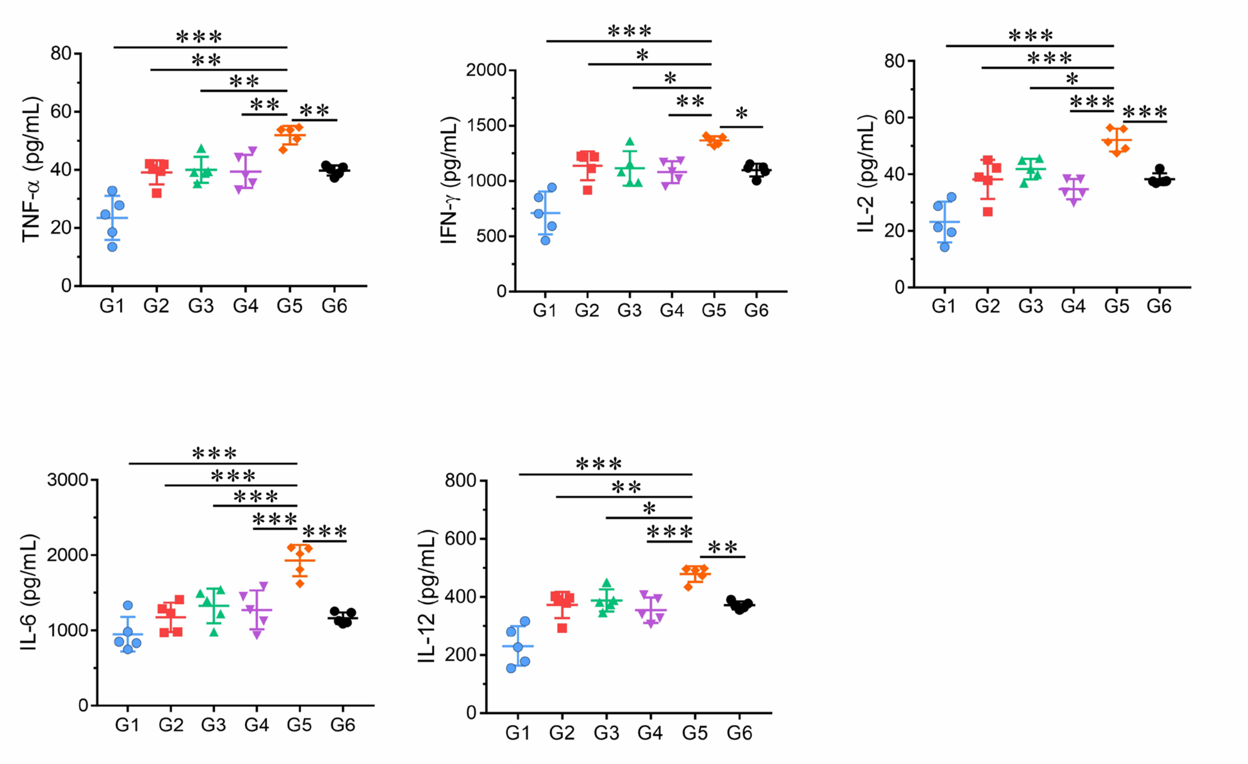

Supplementary Figure 11. Cytokine detection in metastatic lesions after treatment. Cytokine levels in the metastatic lesion within the lung parenchyma collected 7 days after different treatments, $n = 5$ biologically independent animals. G1, PBS; G2, aPDL1; G3, Vadimezan; G4, P@aPDL1; G5, P@aPDL1 + Vadimezan; G6, P@IgG + Vadimezan. Data are presented as mean ± SD, statistical significance is calculated *via* one-way ANOVA with a Tukey post-hoc test. P value: * $P < 0.05$, ** $P < 0.01$, *** $P < 0.001$. TNF-α: G1 *vs.* G5: $P < 0.0001$, G2 *vs.* G5: $P = 0.0015$, G3 *vs.* G5: $P = 0.0031$, G4 *vs.* G5: $P = 0.0019$, G6 *vs.* G5: $P = 0.0025$; IFN-γ: G1 *vs.* G5: $P < 0.0001$, G2 *vs.* G5: $P = 0.0321$, G3 *vs.* G5: $P = 0.0165$, G4 *vs.* G5: $P = 0.0061$, G6 *vs.* G5: $P = 0.0101$; IL-2: G1 *vs.* G5: $P < 0.0001$, G2 *vs.* G5: $P = 0.0008$, G3 *vs.* G5: $P = 0.0132$, G4 *vs.* G5: $P < 0.0001$, G6 *vs.* G5: $P = 0.0008$; IL-6: G1 *vs.* G5: $P < 0.0001$, G2 *vs.* G5: $P < 0.0001$, G3 *vs.* G5: $P = 0.0006$, G4 *vs.* G5: $P = 0.0002$, G6 *vs.* G5: $P < 0.0001$; IL-12: G1 *vs.* G5: $P < 0.0001$, G2 *vs.* G5: $P = 0.0026$, G3 *vs.* G5: $P = 0.011$, G4 *vs.* G5: $P = 0.0005$, G6 *vs.* G5: $P = 0.0025$.

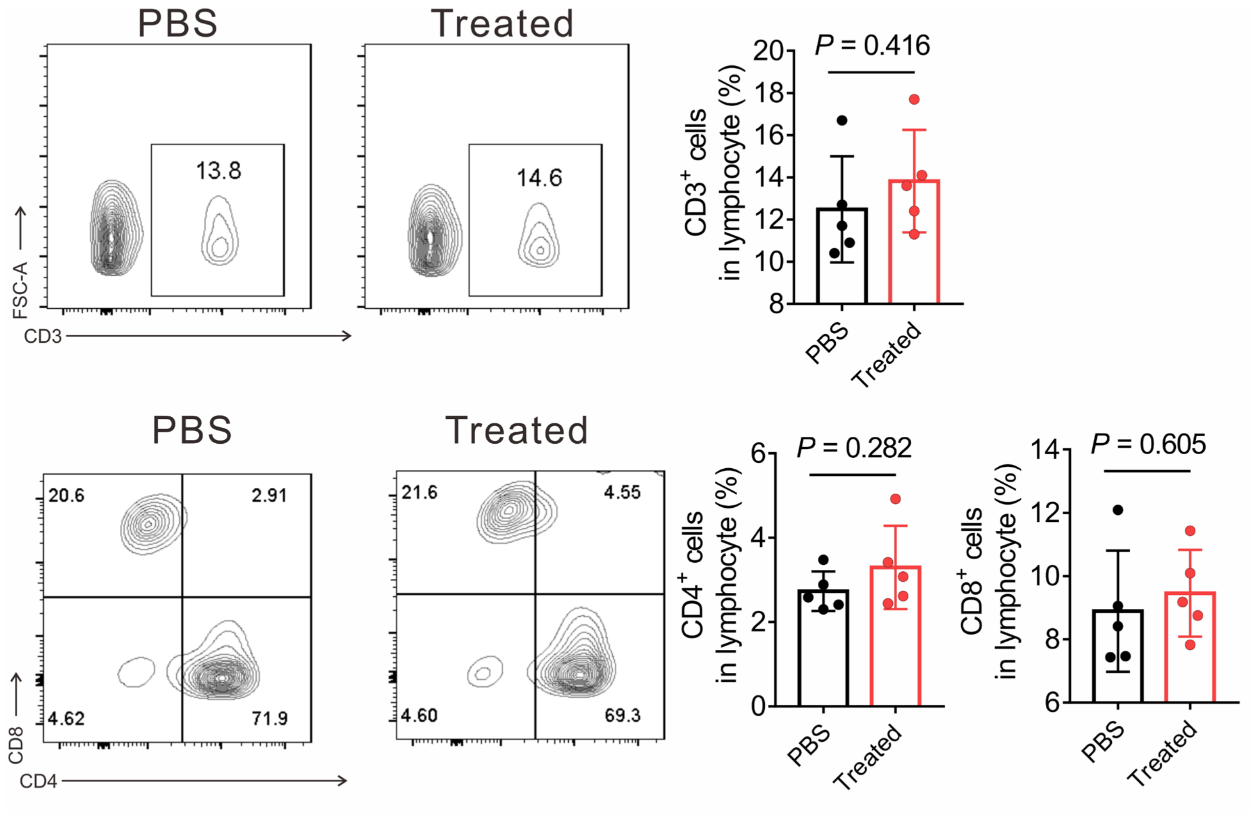

Supplementary Figure 12. Immune response in non-tumour bearing mice after P@aPDL1 + Vadimezan treatment. Representative flow cytometry analysis of $CD3^+$, $CD4^+$ and $CD8^+$ T cells in mice receiving PBS or Vadimezan + platelet@aPDL1 treatment. $n = 5$ biologically independent animals. Data are presented as mean ± SD, statistical significance is calculated via T-test with two-tails.

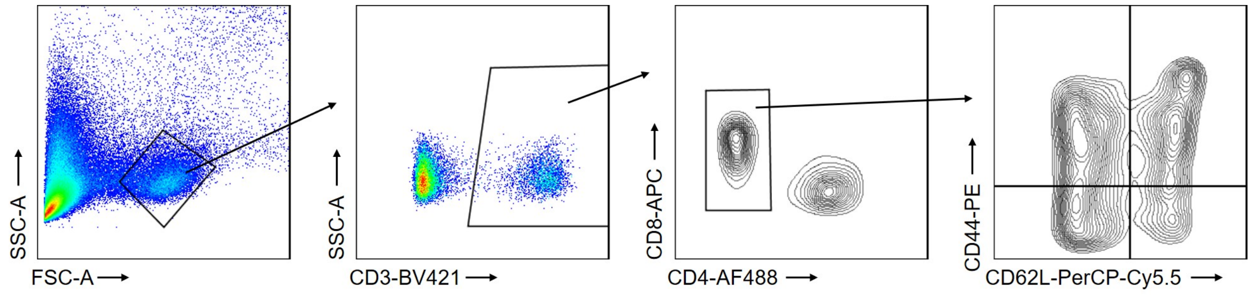

Supplementary Figure 13. Gating strategy to identify T cell memory by flow cytometry.

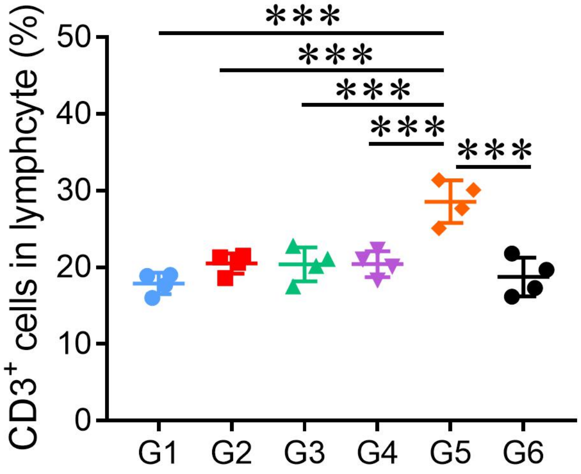

Supplementary Figure 14. CD3$^+$ T cells in the peripheral blood of treated mice. Relative quantification of the CD3$^+$ T cells in the peripheral blood, $n = 4$ biologically independent animals. G1, PBS; G2, aPDL1; G3, Vadimezan; G4, P@aPDL1; G5, P@aPDL1 + Vadimezan; G6, P@IgG + Vadimezan. Data are presented as mean ± SD, statistical significance is calculated *via* one-way ANOVA with a Tukey post-hoc test. *P* value: *** $P < 0.001$. G1 *vs.* G5: $P < 0.0001$, G2 *vs.* G5: $P < 0.0001$, G3 *vs.* G5: $P < 0.0001$, G4 *vs.* G5: $P < 0.0001$, G6 *vs.* G5: $P < 0.0001$.

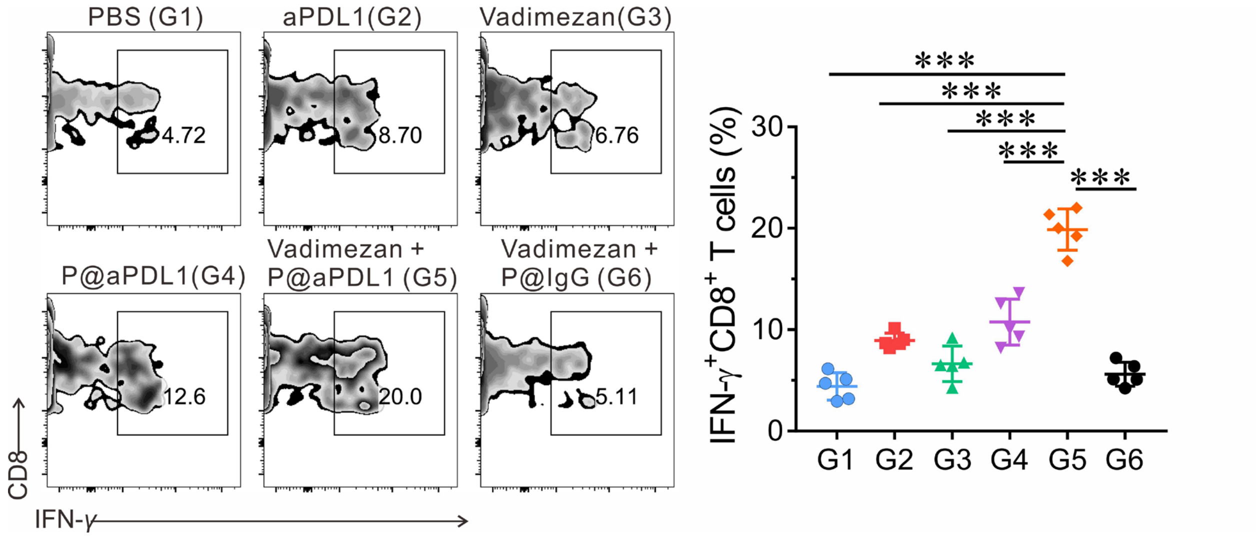

Supplementary Figure 15. Quantification of cytotoxic T cells in B16F10 melanoma metastatic lesions after treatment. Representative flow cytometry analysis of IFN-γ^+CD8$^+$ T cells gating on CD3$^+$ T cells in tumor metastases, n = 5 biologically independent animals. G1, PBS; G2, aPDL1; G3, Vadimezan; G4, P@aPDL1; G5, P@aPDL1 + Vadimezan; G6, P@IgG + Vadimezan. Data are presented as mean ± SD, statistical significance is calculated *via* one-way ANOVA with a Tukey post-hoc test. P value: *** $P < 0.001$. G1 *vs.* G5: $P < 0.0001$, G2 *vs.* G5: $P < 0.0001$, G3 *vs.* G5: $P < 0.0001$, G4 *vs.* G5: $P < 0.0001$, G6 *vs.* G5: $P < 0.0001$.

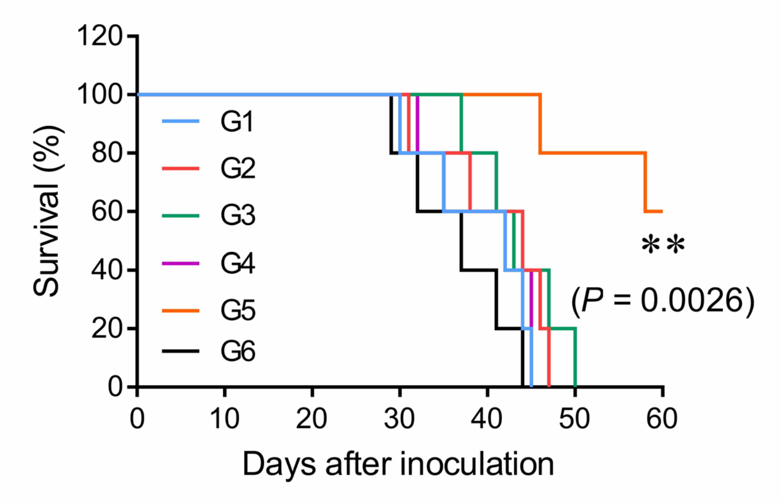

Supplementary Figure 16. Survival of mice bearing metastatic Lewis lung cancer after treatment. The survival cure of mice with metastatic Lewis lung cancer after treatment. Mice received the treatment 10 days after tumor engraftment, $n = 5$ biologically independent animals. G1, PBS; G2, aPDL1; G3, Vadimezan; G4, P@aPDL1; G5, P@aPDL1 + Vadimezan; G6, P@IgG + Vadimezan. Statistical significance of the survival was calculated via log-rank (mantel-cox) test. P value: ** $P < 0.01$.

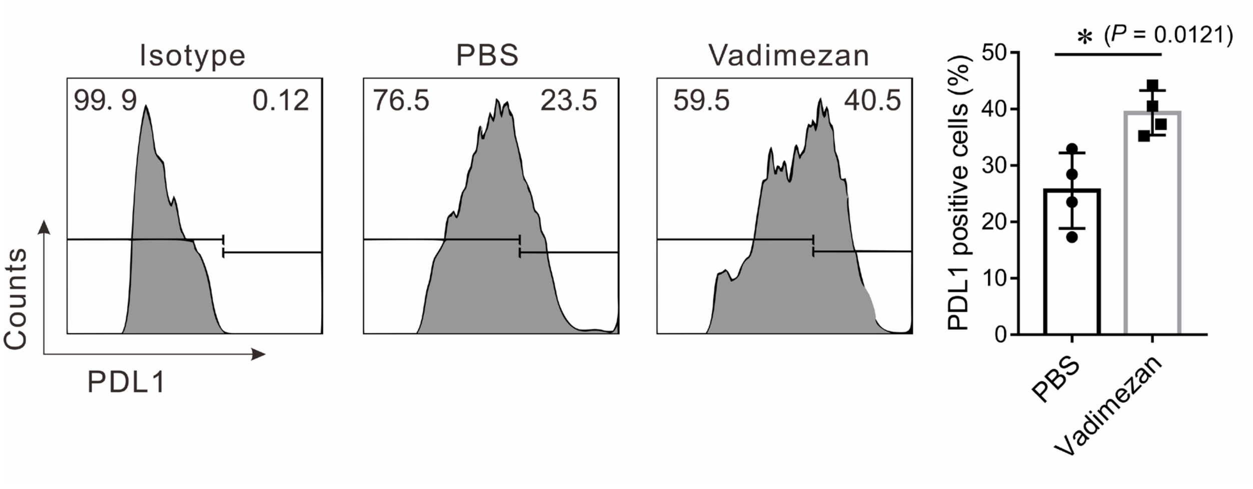

Supplementary Figure 17. PDL1 expression by tumour cells after treatment with Vadimezan. Flow cytometry data showing PDL1 expression in 4T1 breast metastasis after treatment with PBS and Vadizeman. Data are presented as mean ± SD, *n* = 4 biologically independent animals, statistical significance is calculated via one-way ANOVA with a Tukey post-hoc test. *P* value: * $P < 0.05$.
