# Supplementary material for: Disrupting tumour vasculature and recruitment of aPDL1-loaded platelets control tumour metastasis
Source: Nat Commun. 2021 May 13;12:2773. doi: 10.1038/s41467-021-22674-3 (PMC8119987; doi:10.1038/s41467-021-22674-3)
Supplement: Supplementary file 3 — Reporting Summary [file 41467_2021_22674_MOESM3_ESM.pdf]

nature research

Corresponding author(s): Zhen Gu

Last updated by author(s): Feb 23, 2021

Reporting Summary

Nature Research wishes to improve the reproducibility of the work that we publish. This form provides structure for consistency and transparency in reporting. For further information on Nature Research policies, see our Editorial Policies and the Editorial Policy Checklist.

Statistics

For all statistical analyses, confirm that the following items are present in the figure legend, table legend, main text, or Methods section.

n/a	Confirmed	
☐	☒	The exact sample size (*n*) for each experimental group/condition, given as a discrete number and unit of measurement
☐	☒	A statement on whether measurements were taken from distinct samples or whether the same sample was measured repeatedly
☐	☒	The statistical test(s) used AND whether they are one- or two-sided<br>*Only common tests should be described solely by name; describe more complex techniques in the Methods section.*
☒	☐	A description of all covariates tested
☐	☒	A description of any assumptions or corrections, such as tests of normality and adjustment for multiple comparisons
☐	☒	A full description of the statistical parameters including central tendency (e.g. means) or other basic estimates (e.g. regression coefficient) AND variation (e.g. standard deviation) or associated estimates of uncertainty (e.g. confidence intervals)
☐	☒	For null hypothesis testing, the test statistic (e.g. *F*, *t*, *r*) with confidence intervals, effect sizes, degrees of freedom and *P* value noted<br>*Give P values as exact values whenever suitable.*
☒	☐	For Bayesian analysis, information on the choice of priors and Markov chain Monte Carlo settings
☒	☐	For hierarchical and complex designs, identification of the appropriate level for tests and full reporting of outcomes
☒	☐	Estimates of effect sizes (e.g. Cohen's *d*, Pearson's *r*), indicating how they were calculated

Our web collection on statistics for biologists contains articles on many of the points above.

Software and code

Policy information about availability of computer code

Data collection	Zeiss ZEN2, BD FACSDiva 6.1, Live image 4.0
Data analysis	GraphPad Prism 7, ImageJ, FlowJo10.7

For manuscripts utilizing custom algorithms or software that are central to the research but not yet described in published literature, software must be made available to editors and reviewers. We strongly encourage code deposition in a community repository (e.g. GitHub). See the Nature Research guidelines for submitting code & software for further information.

Data

Policy information about availability of data

All manuscripts must include a data availability statement. This statement should provide the following information, where applicable:
- Accession codes, unique identifiers, or web links for publicly available datasets
- A list of figures that have associated raw data
- A description of any restrictions on data availability

All the other data supporting the findings of this study are available within the article and its supplementary information files and from the corresponding author upon reasonable request. Source data are provided with this paper.

Field-specific reporting

April 2020

Life sciences study design

All studies must disclose on these points even when the disclosure is negative.

Sample size	The sample size were determined as per the pilot study or previous experimental experience and standard protocols in the field (Huo. et al., 2019, Nat Comms; Zhao. et al., 2018, Nat Comms)
Data exclusions	No data were excluded.
Replication	The experimental findings were reliably reproduced. All of the studies are repeated at two times.
Randomization	All samples/organisms were randomly allocated into experimental groups.
Blinding	No formal blinding was used. The investigator organizing the experimental groups and involved in sample collection was not blinded; however, colleagues aiding in data collection were blinded.

Reporting for specific materials, systems and methods

We require information from authors about some types of materials, experimental systems and methods used in many studies. Here, indicate whether each material, system or method listed is relevant to your study. If you are not sure if a list item applies to your research, read the appropriate section before selecting a response.

Materials & experimental systems

n/a	Involved in the study	
☐	☒	Antibodies
☐	☒	Eukaryotic cell lines
☒	☐	Palaeontology and archaeology
☐	☒	Animals and other organisms
☒	☐	Human research participants
☒	☐	Clinical data
☒	☐	Dual use research of concern

Methods

n/a	Involved in the study	
☒	☐	ChIP-seq
☐	☒	Flow cytometry
☒	☐	MRI-based neuroimaging

Antibodies

Antibodies used	The anti-mouse PDL1 antibody was purchased from BioLegend Inc. (catalog no. 124329, clone: 10F.9G2). The antibodies used for flow cytometry analysis and immunofluorescence staining including: CD3 (BioLegend, catalog no. 100336, clone: 145-2C11, Brilliant Violet 421 -conjugated), CD4 (BioLegend, catalog no. 100408, clone: GK1.5, PE -conjugated), CD4 (BioLegend, catalog no. 100406, clone: GK1.5, FITC -conjugated), CD8 (Invitrogen, catalog no. 2023410, clone: 53-6.7, APC -conjugated), Ki67 (BioLegend, catalog no. 652410, clone: 16A8, FITC -conjugated), IFN-γ (BioLegend, catalog no. 505806, clone: XMG1.2, FITC -conjugated), CD44 (BioLegend, catalog no. 103024, clone: 1M7, PE -conjugated), CD62L (BioLegend, catalog no. 304823, clone: DREG-56, PerCP/Cyanine5.5 -conjugated), CD9 (BioLegend, catalog no. 124807, clone: MZ3, FITC -conjugated), CD41 (BioLegend, catalog no. 133904, clone: MWReg30, PE -conjugated), CD36 (BioLegend, catalog no. 102605, clone: HM36, PE -conjugated), CD61 (BioLegend, catalog no. 104307, clone: 2C9.G2, PE -conjugated), CD62P (BioLegend, catalog no. 148305, clone: RMP-1, PE -conjugated), CD154 (BioLegend, catalog no. 106505, clone: MR1, PE -conjugated), PDL1 (BioLegend, catalog no. 124308, clone: 10F.9G2, PE -conjugated), CD62P (R&D Systems, catalog no. AF737, goat IgG), CD31 (R&D Systems, catalog no. AF3628, goat IgG), Donkey anti goat IgG (Invitrogen, catalog no. 2044862, AF568 -conjugated), goat anti rat IgG (Invitrogen, catalog no. 2040140, APC -conjugated).
Validation	All antibodies are commercially available. Antibodies employed here in our manuscript were previously reported and routinely used for the application used. All companies used report quality control measures to ensure validity and reproducibility. Validation information and previous citations for each individual antibody are found in the data sheets provided by the company.

Eukaryotic cell lines

Policy information about cell lines

Cell line source(s)	4T1, 4T1-Luc, B16F10, B16F10-Luc, Lewis lung carcinoma were purchased from ATCC.
Authentication	All of the cells are purchased form ATCC, and was not authenticated.
Mycoplasma contamination	It is negative for mycoplasma
Commonly misidentified lines (See ICLAC register)	No commonly misidentified cell lines were used in the study.

Animals and other organisms

Policy information about studies involving animals; ARRIVE guidelines recommended for reporting animal research

Laboratory animals	All of the Balb/B C mice, C57 mice were female in 5-7 weeks old.
Wild animals	The study did not involve wild animals.
Field-collected samples	The study did not involve samples collected from the field.
Ethics oversight	All mouse studies were carried out under the protocols approved by the Institutional Animal Care and Use Committee at the University of California, Los Angeles (UCLA).

Note that full information on the approval of the study protocol must also be provided in the manuscript.

Flow Cytometry

Plots

Confirm that:

- [x] The axis labels state the marker and fluorochrome used (e.g. CD4-FITC).
- [x] The axis scales are clearly visible. Include numbers along axes only for bottom left plot of group (a 'group' is an analysis of identical markers).
- [x] All plots are contour plots with outliers or pseudocolor plots.
- [x] A numerical value for number of cells or percentage (with statistics) is provided.

Methodology

Sample preparation	Blood samples were harvested (breast metastasis, 7 days; melanoma metastasis, 14 days) post-treatment. The cells were stained with antibodies post lysis process to remove the red blood cells, all of the antibodies stainings was followed the manufacturer's instructions.
Instrument	BD LSR II flow cytometer
Software	BD FACSDiva
Cell population abundance	The instrument counts 10,000 or 50,000 cells autonomously.
Gating strategy	The gating strategies are displayed in the supplementary information.

- [x] Tick this box to confirm that a figure exemplifying the gating strategy is provided in the Supplementary Information.
